# Obesogenic Diet Cycling Produces Graded Effects on Cognition and Microbiota Composition in Rats

**DOI:** 10.1002/mnfr.202200809

**Published:** 2023-05-05

**Authors:** Michael D. Kendig, Sarah‐Jane Leigh, Kyoko Hasebe, Nadeem O. Kaakoush, R. Fred Westbrook, Margaret J. Morris

**Affiliations:** ^1^ School of Medical Sciences UNSW Sydney Sydney NSW 2052 Australia; ^2^ School of Life Sciences University of Technology Ultimo NSW 2007 Australia; ^3^ APC Microbiome University of Cork Cork T12 K8AF Ireland; ^4^ School of Psychology UNSW Sydney Sydney NSW 2052 Australia

**Keywords:** cafeteria diet, cognitive impairment, diet cycling, gut microbiota, obesity

## Abstract

**Scope:**

The effects of diet cycling on cognition and fecal microbiota are not well understood.

**Method and Results:**

Adult male Sprague‐Dawley rats were cycled between a high‐fat, high‐sugar “cafeteria” diet (Caf) and regular chow. The impairment in place recognition memory produced by 16 days of Caf diet was reduced by switching to chow for 11 but not 4 days. Next, rats received 16 days of Caf diet in 2, 4, 8, or 16‐day cycles, each separated by 4‐day chow cycles. Place recognition memory declined from baseline in all groups and was impaired in the 16‐ versus 2‐day group. Finally, rats received 24 days of Caf diet continuously or in 3‐day cycles separated by 2‐ or 4‐day chow cycles. Any Caf diet access impaired cognition and increased adiposity relative to controls, without altering hippocampal gene expression. Place recognition and adiposity were the strongest predictors of global microbiota composition. Overall, diets with higher Caf > chow ratios produced greater spatial memory impairments and larger shifts in gut microbiota species richness and beta diversity.

**Conclusion:**

Results suggest that diet‐induced cognitive deficits worsen in proportion to unhealthy diet exposure, and that shifting to a healthy chow for at least a week is required for recovery under the conditions tested here.

## Graphical abstract

The effects of cycling between a high‐fat, high‐sugar “cafeteria” diet (Caf) and chow on cognition, adiposity, and gut microbiota composition in adult male rats were tested. Diets with higher Caf:chow ratios produced greater spatial memory impairments (left) and shifts in beta diversity (right). Our results show “dose‐dependent” effects of unhealthy diets on cognition and gut microbiota composition, even over relatively short‐term diet exposure.

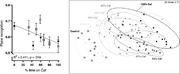

## 1 Introduction

Highly‐processed “junk” foods rich in fat and sugar are a central part of modern diets^[^ ^1^ ^]^ and increase the risk of obesity and adverse health effects.^[^ ^2^ ^]^ However, these foods are seldom eaten exclusively: people may alternate between periods of healthy and unhealthy eating in so‐called “yo‐yo dieting” or weight cycling,^[^ ^3^ ^]^ and diet quality tends to be poorer on weekends,^[^ ^4^, ^5^, ^6^ ^]^ linked to body weight changes.^[^ ^7^ ^]^ It is therefore important to identify the effects of intermittent junk food consumption on longer‐term outcomes.

Intermittent access to diets high in fat and/or sugar can induce “binge‐like” consumption and increase body weight and adiposity in rodents.^[^ ^8^, ^9^, ^10^, ^11^, ^12^ ^]^ Alternating 2‐week cycles of low‐ and high‐fat diets produced similar increases in adiposity and impairments in glucose tolerance as continuous high‐fat diet in mice^[^ ^13^ ^]^ while cycling between low‐ and high‐fat diets for 4‐week periods exhibited increased adiposity without affecting lifespan compared to mice fed low‐fat diet.^[^ ^14^ ^]^ Cycling access to a high‐sucrose diet in rats produced persistent responding for palatable food rewards despite punishment^[^ ^15^ ^]^ but suppressed responding for standard chow.^[^ ^8^, ^16^ ^]^

High‐fat, high‐sugar diets are also associated with poorer cognition in human and rodents^[^ ^17^, ^18^ ^]^ but the cognitive effects of diet cycling are not well understood. On the one hand, the beneficial effects on cognition produced by intermittent fasting^[^ ^19^, ^20^ ^]^ or by switching from an unhealthy to healthy diet^[^ ^21^, ^22^ ^]^ suggest that cognition may be preserved in diet cycling. On the other hand, high‐fat, high‐sugar diets can impair cognition within days in humans and rodents,^[^ ^23^, ^24^, ^25^, ^26^ ^]^ suggesting that even relatively brief exposure poses risk.

The present study explored the cognitive effects of diet cycling by systematically manipulating access to healthy and unhealthy diets in an established rat model.^[^ ^12^ ^]^ Changes in short‐term recognition memory were evaluated alongside metabolic measures, as well as gut microbiota composition which is associated with variations in cognition^[^ ^27^ ^]^ and sensitive to shifts in diet.^[^ ^28^, ^29^ ^]^

We recently found that 3 days per week access to a palatable high‐fat, high‐sugar cafeteria‐style (Caf) diet was sufficient to alter gut microbiota composition^[^ ^30^, ^31^ ^]^ without the impairment in place recognition memory observed following 5 or 7 days per week access.^[^ ^32^ ^]^ Relative to the groups fed Caf diet for 5 or 7 days per week, intact cognition in the group fed Caf diet for 3 days per week could be explained either by their shorter weekly exposure to Caf diet (3 days), or their longer weekly access to chow diet (4 days) providing greater respite from the Caf diet. To contrast these possibilities, the present study first tested whether a single 4‐day chow cycle could restore short‐term memory after continuous Caf diet. The next two experiments systematically varied Caf and chow cycle length, respectively, to determine their relative effects on cognition and adiposity during diet cycling. If Caf cycle length is critical, longer Caf cycles should produce greater diet‐induced impairments; to test this, we compared 2, 4, 8, and 16‐day Caf cycles, each separated by 4‐day chow cycles. By contrast, if chow cycle length is critical, shorter chow cycles should produce greater diet‐induced impairments. To test this, we compared 3‐day Caf cycles that were separated by 2‐ or 4‐day chow cycles, alongside groups fed continuous chow or Caf. Changes in short‐term recognition memory, whole‐body adiposity, hippocampal gene expression, and gut microbiota composition were assessed.

## 2 Experimental Section

Experimental procedures were approved by the Animal Care and Ethics Committee at the University of New South Wales (ethics #17/65A) and complied with the guidelines for the use and care of animals for scientific purposes 8th edition (National Health and Medical Research Council, Australia).

### 2.1 Subjects

Subjects were male Sprague‐Dawley rats (*n* = 144; Animal Resource Centre, Perth, Australia), weighing 185–220 g on arrival to the laboratory. They were housed three per cage (47 × 29 × 15 cm) in a temperature‐ and humidity‐controlled colony room (lights off 1500–0300 h). Chow (14.2 kJ g^−1^, 65% carbohydrate, 23% protein, 12% fat, Gordon's Irradiated Rat and Mouse Diet) and potable water were available to all groups ad libitum. Rats were handled and weighed regularly during a week‐long acclimation, followed by baseline short‐term recognition memory tests, and were then allocated to diet groups matched for body weight and recognition memory performance. Prior to the diet intervention, mixed feces collected from each cage were redistributed to standardize the gut microbiome. One rat in Experiment 1 became unwell prior to the Caf diet intervention and was euthanized.

### 2.2 Cafeteria Diet and Food Intake Measures

The cafeteria diet (Caf) consisted of a variety of commercially produced processed foods rich in fat and sugar, plus a 10% sucrose solution, provided in addition to chow and water, and replenished daily.^[^ ^33^ ^]^ Continuous and cycled Caf groups received the same menu. Food intake per cage (*n* = 4 cages per group) was measured by weighing food and bottles of water and sucrose at the beginning of the dark cycle and 24‐h later; consumption in grams was converted to kJ using product information.

### 2.3 Place and Object Recognition Memory Tests

Recognition memory was assessed between 0700 and 1400 h in an arena as described previously.^[^ ^31^, ^32^ ^]^ Rats were familiarized with the black acrylic arena (60 × 60 × 60 cm) in two 5‐min sessions prior to their first test and once prior to subsequent tests. Place and object tests consisted in 5‐min exposure to two identical objects (glass jars, aluminum tins, etc.) in the center of the arena. The rat was returned to its home‐cage for 5‐min while the arena and objects were cleaned with 50% ethanol, then placed in the arena for a 3‐min test with one novel and one familiar object (object test) or the two original objects with one moved to a novel location (place test). Tests were recorded and a trained observer “blind” with respect to group scored exploration, excluding rats that explored either object for less than 2 s (one rat in Experiment 3).

### 2.4 Experimental Design

**Figure 1:**
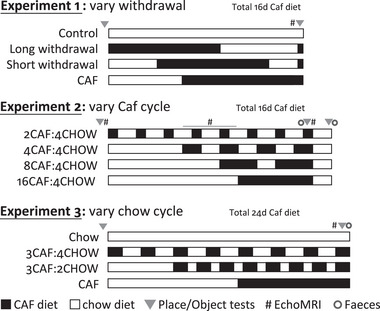
Experimental design. The four groups in Experiment 1 received chow (control) or 16 days of Caf diet, with two groups withdrawn to chow for 4 or 11 days. The four groups in Experiment 2 received 16 days of Caf diet in 2, 4, 8, or 16‐day cycles, with each cycle separated by 4 days of chow. The four groups in Experiment 3 received chow (control), or 24 days of Caf diet provided continuously, or in 3‐day cycles separated by 4 or 2 days of chow. The introduction of Caf diet was staggered so that final tests could be conducted at the same time across groups.

**Figure** **1** shows the design of the three experiments, which each used four groups of 12 rats. The introduction of Caf diet cycling was staggered so that final tests and tissue collection were conducted concurrently; place and object recognition were assessed at baseline and at the end of each experiment. Experiment 1 tested whether withdrawal from Caf to chow rescued short‐term place recognition memory. A control group fed chow was compared with two groups fed Caf diet for 16 days and then withdrawn to chow for 11 (Long withdrawal), or 4 (Short withdrawal) days; the final group was fed Caf diet for 16 continuous days (CAF). Long and Short withdrawal groups were returned to Caf diet for ≈36 hours prior to the second test in order to compare their performance while on the diet to that of group CAF. Experiment 2 varied Caf cycle length while holding chow cycle length constant across groups. Four groups were fed Caf diet for a total of 16 days in 2‐, 4‐, 8‐, or 16‐day cycles, each separated by 4‐day chow cycles (2CAF:4CHOW, 4CAF:4CHOW, 8CAF:4CHOW, and 16CAF:4CHOW groups, respectively). Experiment 3 varied chow cycle length holding Caf cycle length constant across groups. A chow‐fed control group was compared to three groups fed Caf diet for a total of 24 days, provided continuously (CAF group) or in 3‐day cycles separated by 2 or 4 days (3CAF:2CHOW and 3CAF:4CHOW groups, respectively).

### 2.5 Adiposity and Endpoint Measures

Whole‐body fat and lean mass were measured by EchoMRI (BRIL, Mark Wainwright Analytical Centre, UNSW Sydney). In Experiments 2 and 3, rats were deeply anesthetized (ketamine/xylazine, i.p.), and then decapitated; anthropometric measures, liver and retroperitoneal fat pad mass were recorded, and a single fecal pellet was collected from the descending colon and snap‐frozen.

### 2.6 Fecal DNA Extraction and 16S rRNA Gene Sequencing

In Experiments 2 and 3, feces were collected at the end of Caf diet exposure, DNA extracted (DNeasy PowerSoil Pro kit, Qiagen, 47016) and subjected to amplification and Illumina sequencing (2 × 250 bp MiSeq chemistry, V4 region, 515F‐806R primer pair; Ramaciotti Centre for Genomics, UNSW Sydney). Mothur (v.1.44.2)^[^ ^34^ ^]^ was used to process the resulting sequence data with commands modified from the MiSeq SOP^[^ ^35^ ^]^ to align with the SILVA database (v128), remove singletons, check chimeras with UCHIME, and classify with the RDP training set (version16_022016). Sequence data were subsampled to *n* = 4516 clean reads per sample.

### 2.7 Data Analysis

Data were analyzed with IBM SPSS Statistics (v26) using one‐way ANOVAs followed by post‐hoc pairwise comparisons applying the Tukey HSD correction. Nonparametric Kruskal–Wallis tests were used for data that were not normally distributed. Mixed‐ANOVAs were used to analyze energy intake, with cage as unit of analysis. Fecal microbiota data were analyzed using Primer v7^[^ ^36^ ^]^ to calculate alpha diversity measures and a Bray–Curtis similarity matrix at the OTU level, calculated on relative abundances following a square root transformation. Group differences in microbiota composition were assessed by simultaneous multiple regression (see Supplementary Figure S4), permutational multivariate ANOVA (PERMANOVA), and in nonmetric multidimensional scaling plots. Distance‐based linear modeling was used to identify which phenotypic measures were associated with the OTU similarity matrix. Housing cage (*n* = 3 rats per cage) was factored into microbiota analyses as a covariate. All results are expressed as mean ± SEM. Results were considered significant when *p* < 0.05.

## 3 Results

### 3.1 Experiment 1: Caf Diet Withdrawal Produces Time‐Sensitive Recovery of Place Recognition Memory Impairments

**Figure 2:**
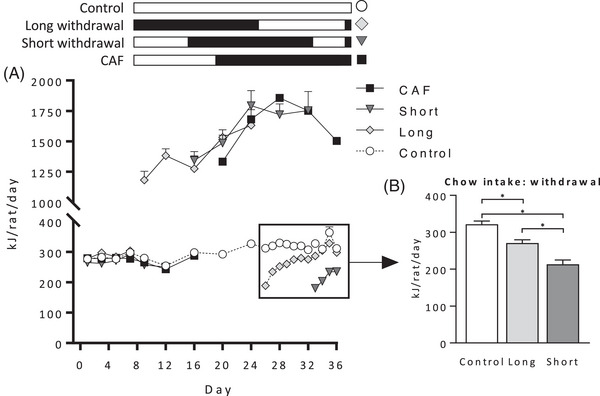
Energy intake in Experiment 1. The schematic indicates when groups were exposed to Caf (black) or chow (open) diets. Long, Short, and CAF groups received 16 days of Caf diet, with groups Long and Short subsequently withdrawn to chow for 11 (Long) or 4 days (Short). A) Caf diet increased energy intake relative to controls. B) Average daily chow intake after Caf withdrawal was lower in groups Long and Short than the control group. *n* = 4 cages per group. Data analyzed by one‐way analysis of variance (ANOVA) followed by Tukey post‐hoc; *p < 0.05.

As shown in **Figure** **2**, Caf diet exposure significantly increased energy intake in groups Long, Short, and CAF relative to Control (ANOVA: *F*(3, 12) = 122.55, *p* < 0.001, Tukey post‐hoc all *p* < 0.01) with no significant differences between the Caf‐fed groups (all *p* > 0.05). During withdrawal to chow, average daily intake was significantly lower in groups Long and Short than Control, and significantly lower in group Long than Short (ANOVA: *F*(2, 9) = 31.76, *p* < 0.001, Tukey post‐hoc: Control versus Long *p* = 0.012, Control versus Short *p* < 0.001; Long versus Short *p* = 0.006).

### 3.2 Body Weight

**Figure 3:**
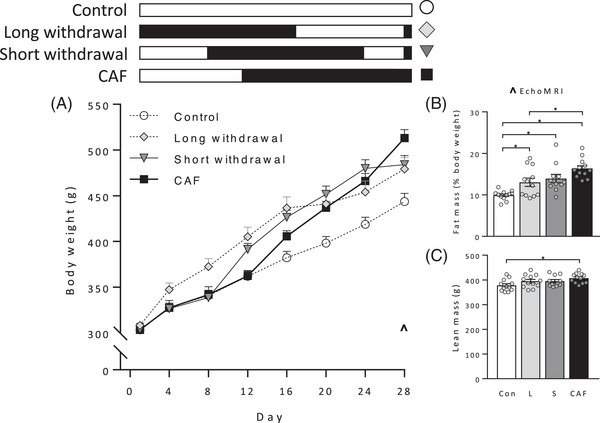
Body weight gain, fat, and lean mass in Experiment 1. The schematic indicates when groups were exposed to Caf (black) and chow (white) diets. Long, Short, and CAF groups received 16 days of Caf diet, with groups Long and Short subsequently switched to chow for 11 (Long) or 4 days (Short). Body weight A) and percent fat mass B) were significantly greater in group CAF and group Short than Controls, and intermediate in group Long. Lean mass C) was significantly elevated in group CAF relative to group Control. *n* = 11–12 per group. The ^ symbol in panel A denotes timing of body composition assessed by EchoMRI. Data were analyzed by one‐way analysis of variance (ANOVA) followed by Tukey post‐hoc; *p < 0.05.

Body weight at the end of withdrawal differed significantly between groups (**Figure** **3**a; *F*(3, 43) = 7.58, *p* < 0.001) and was significantly greater in CAF and Short groups than controls (*p* = 0.046, *p* < 0.001, respectively). Percent fat mass was elevated in all Caf‐fed groups relative to controls (Figure 3b; *F*(3, 43) = 11.26, *p* < 0.001; Tukey post‐hoc, all *p* < 0.05) and significantly lower in group Long than group CAF (*p* = 0.025). Net lean mass was greater in the CAF group relative to control (Figure 3c; *F*(3, 43) = 3.28, *p* = 0.03; Tukey post‐hoc: *p* = 0.017), with no other group differences.

### 3.3 Place and Object Recognition

**Figure 4:**
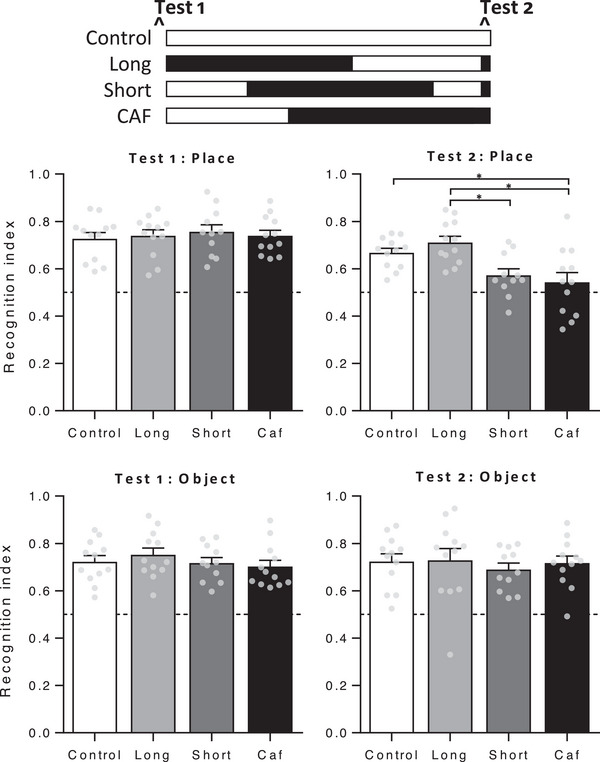
Place and object recognition in Experiment 1. The schematic indicates when groups were exposed to Caf (black) and chow (white) diets. Baseline tests (left panels) showed no group differences in performance on either test. Test 2 was held after 16 days of Caf diet and withdrawal to chow for 11 (Long) or 4 (Short) days. Place recognition memory (top right) was impaired in groups Short and CAF relative to group Long, and in group CAF relative to controls, with no difference in object recognition (bottom right). *n* = 11–12 per group. Dashed line at 0.5 indicates impaired short‐term memory; i.e., equal exploration of both objects. Data were analyzed by one‐way analysis of variance (ANOVA) followed by Tukey post‐hoc; *p < 0.05.

Groups did not differ in place recognition at baseline (**Figure** **4**, left, *F* < 1). At Test 2, place recognition differed across groups (*F*(3, 43) = 7.20, *p* = 0.001) and Tukey post‐hoc comparisons confirmed the expected impairment in group CAF relative to controls (*p* = 0.022). Notably, place recognition was significantly better in group Long than groups CAF and Short (*p* = 0.001 and *p* = 0.011, respectively) and did not differ significantly from controls (*p* = 0.72). No group differences were found in perirhinal cortex‐dependent object recognition at either test (both *F* < 1), as expected. Thus, withdrawal from Caf diet rescued a selective impairment in short‐term place recognition memory in a time‐dependent fashion, with performance significantly improved in rats withdrawn to chow for 11 but not 4 days. Importantly, the memory impairment in Short and CAF groups was not due to differences in motivation to engage with the task, as total object exploration time did not differ between groups on either test (both *F* < 1).

### 3.4 Experiment 2: Longer Caf Diet Cycles Worsen Place Recognition Memory Impairments

Experiment 2 tested short‐term place recognition memory in rats cycled between Caf and chow diets, where the chow cycle duration was fixed at 4 days and the Caf cycle duration was systematically varied. Four groups received 16 days total exposure to Caf diet in cycles of 2, 4, 8, or 16 days (Figure 1). Place recognition memory was tested at baseline, on Caf day 16, and 4 days later on chow, to separate any acute effects of switching diets from the cumulative effects of Caf diet intake.

### 3.5 Energy Intake

**Figure 5:**
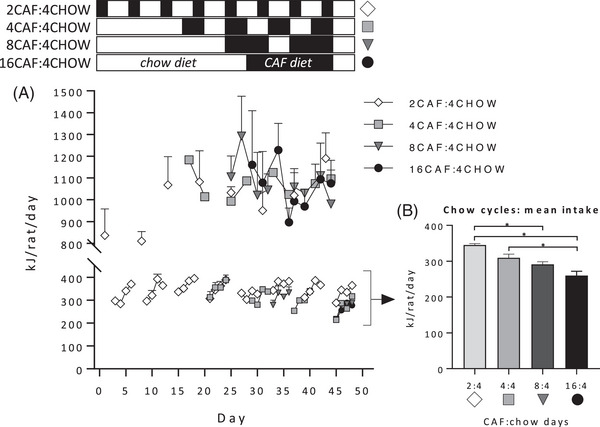
Energy intake in Experiment 2. The schematic indicates when groups were exposed to Caf (black) and chow (white) diets. The four groups received 16 days of Caf diet in 2, 4, 8, or 16‐day blocks, separated by 4 days on chow. A) Energy intake on Caf diet days did not differ significantly between groups. B) Energy intake during chow cycles was significantly lower for 8CAF:4CHOW and 16CAF:4CHOW groups relative to 2CAF:4CHOW and 4CAF:4CHOW groups, averaged across the experiment. *n* = 4 cages per group. Data were analyzed by one‐way analysis of variance (ANOVA) followed by Tukey post‐hoc; *p < 0.05.

Energy intake on Caf diet days (**Figure** **5**a) did not differ according to cycling schedule, with a 4 x (8) mixed‐ANOVA revealing no significant effects of time (linear trend: *F* < 1), group (*F* < 1), and no time x group interaction (*F*(1, 12) = 3.00, *p* = 0.073). Given that the number of chow cycles differed between groups, we calculated average intake on chow cycle days 1, 2, 3, and 4 and analyzed using a 4 x (4) mixed‐ANOVA (group x [day]). This indicated that energy intake increased across the 4‐day chow cycle (“day” linear trend: *F*(1, 12) = 61.16, *p* < 0.001), and differed between groups (*F*(3, 12) = 15.82, *p* < 0.001), with significantly higher chow intake in group 2CAF:4CHOW than groups 8CAF:4CHOW and 16CAF:4CHOW, and in group 4CAF:4CHOW than group 16CAF:4CHOW (Figure 5b; all *p* ≤ 0.01).

### 3.6 Weight Gain and Body Composition

**Figure 6:**
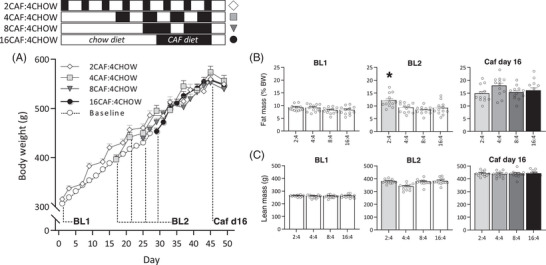
Body weight gain, fat, and lean mass in Experiment 2. The schematic indicates when groups were exposed to Caf (black) and chow (white) diets. The four groups received 16 days of Caf diet in 2, 4, 8, or 16‐day blocks, separated by 4 days on chow. There were no group differences in body weight A), percent fat mass B), or lean mass C) on Caf day 16. *n* = 12 per group. Data were analyzed by one‐way analysis of variance (ANOVA); “Baseline” in panel A refers to the rats yet to commence Caf diet cycling. BL1, time of first baseline EchoMRI measure; BL2, time of second EchoMRI measure, taken on Caf day 8 for group 2CAF:4CHOW and prior to Caf day 1 for other groups. *p < 0.05 versus other groups; Tukey post‐hoc.

The trajectory of body weight gain was comparable across groups, with no significant group differences on Caf day 16 (**Figure** **6**a, *F* < 1). While groups did not differ in percent fat mass or net lean mass at baseline (“BL1”; Figure 6b,c), a second measure taken on Caf day 8 for the 2CAF:4CHOW group confirmed fat mass was significantly elevated relative to other groups yet to commence Caf cycling (*F*(3, 44) = 6.77, *p* = 0.001; Tukey post‐hoc, all *p* < 0.015). By Caf day 16, however, there were no differences in fat (*F* < 1) or lean (*F*(3, 44) = 1.74, *p* = 0.17) mass across groups, nor in terminal plasma hormone, adiposity, or anthropometric measures (Supplementary Table S1), indicating that the schedules produced comparable metabolic phenotypes.

### 3.7 Place Recognition

**Figure 7:**
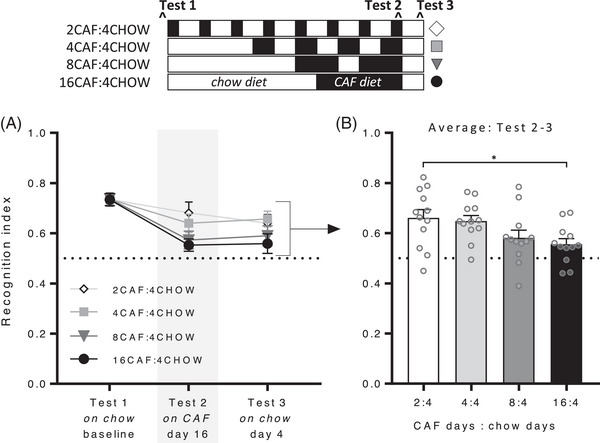
Place recognition memory in Experiment 2. The schematic indicates when groups were exposed to Caf (black) and chow (white) diets. The four groups received 16 days of Caf diet in 2, 4, 8, or 16‐day blocks, separated by 4 days on chow. A) Place recognition memory declined significantly in all groups from baseline to Caf day 16, and was stable 4 days later on chow. B) Averaged across Tests 2 and 3, place recognition memory was significantly lower in group 16CAF:4CHOW than 2CAF:4CHOW, and not significantly different in other groups. *n* = 12 per group. Dashed line at 0.5 indicates equal exploration of both objects and an impairment in short‐term memory. Data were analyzed by one‐way analysis of variance (ANOVA) followed by Tukey post‐hoc; *p < 0.05.

Place recognition memory decreased significantly over tests (**Figure** **7**a; linear trend *F*(1, 44) = 40.11, *p* < 0.001) and did not vary across groups (time x group interaction; *F*(3, 44) = 1.31, *p* = 0.29). When averaged across tests 2 and 3, place recognition was poorer in group 16CAF:4CHOW than group 2CAF:4CHOW (*F*(3, 44) = 3.44, *p* = 0.025; Tukey post‐hoc, *p* = 0.046) with no other significant pairwise comparisons, and no overall difference between performance on test 2 (on Caf) and test 3 (on chow) (*F* < 1) nor in total exploration time (*F*(3, 44) = 2.09, *p* = 0.12). Caf diet cycle length thus modulated place recognition memory in a graded fashion, with impaired performance following longer relative to shorter cycles, controlling for total days of Caf diet and chow cycle duration. These effects were observed despite no group differences in adiposity, which increased from baseline to a comparable extent in all groups, ultimately reaching similar values as Caf‐fed groups in Experiment 1.

### 3.8 Experiment 3: Place Recognition Memory Impairments Are Unaffected by Manipulating Chow Cycle Duration

To test whether chow cycle length is an important determinant of Caf diet‐induced cognitive impairment, Experiment 3 held Caf cycle length constant at 3 days and varied chow cycle length at 2 or 4 days, with 24 days total Caf diet exposure. This duration of Caf diet exposure was chosen because our previous studies indicate that cycling between 3 day Caf/4 day chow does not impair place recognition after 23–25 days of Caf access.^[^ ^31^, ^32^ ^]^ Thus, if chow cycle length mediates recovery and/or protection from short‐term memory impairments, place recognition memory should be higher following 4‐ versus 2‐day chow cycles.

### 3.9 Energy Intake

**Figure 8:**
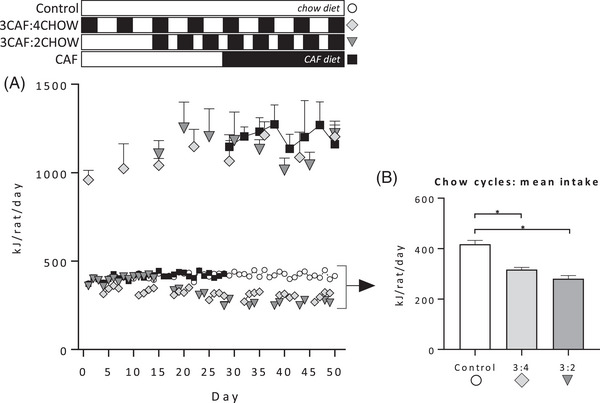
Energy intake in Experiment 3. The schematic indicates when groups were exposed to Caf (black) and chow (white) diets. Control rats fed chow were compared with groups fed Caf diet for 24 days continuously (CAF) or in 3‐day cycles separated by 4 days (3CAF:4CHOW) or 2 days (3CAF:2CHOW) of chow. A) Caf diet intake, measured on the first day of each Caf cycle (or equivalent), was elevated in all Caf‐fed groups relative to chow, with no differences between continuous and cycled groups. B) Cycled groups suppressed energy intake during chow cycles relative to controls fed chow. *n* = 4 cages per group. Data were analyzed by one‐way analysis of variance (ANOVA) followed by Tukey post‐hoc; *p < 0.05.

Energy intake when Caf diet was available was significantly higher in all three Caf‐fed groups relative to controls (**Figure** **8**a; ANOVA: *F*(3, 12) = 32.02, *p* < 0.001; Tukey post‐hoc all *p* < 0.001) and did not change over time (*F*(1, 12) = 2.91, *p* = 0.11). During chow cycles, groups 3CAF:4CHOW and 3CAF:2CHOW significantly suppressed energy intake relative to the chow‐fed control group (Figure 8b; ANOVA: *F*(2, 9) = 31.42, *p* < 0.01, Tukey post‐hoc tests both *p* < 0.01 versus control).

### 3.10 Weight Gain and Fat Mass

**Figure 9:**
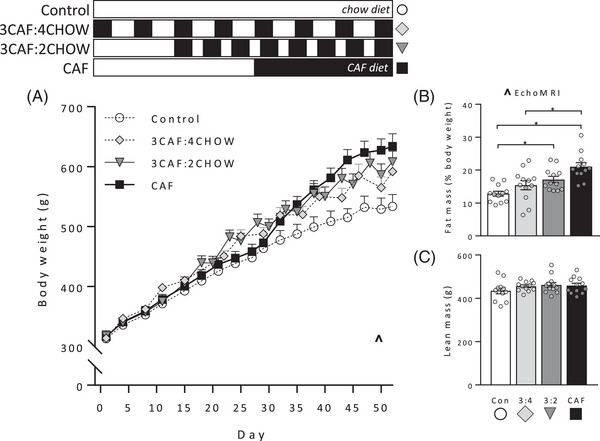
Body weight and adiposity in Experiment 3. The schematic indicates when groups were exposed to Caf (black) and chow (white) diets. Control rats fed chow were compared with groups fed Caf diet for 24 days continuously (CAF) or in 3‐day cycles separated by 4‐day (3CAF:4CHOW) or 2‐day (3CAF:2CHOW) chow cycles. Terminal body weight A) and percent fat mass B) were significantly greater in CAF and 3CAF:2CHOW groups than the control group, while group 3CAF:4CHOW did not differ from the 3CAF:2CHOW or control groups. C) lean mass did not vary between groups. *n* = 12 per group. The ^ symbol in panel A denotes the timing of body composition assessment by EchoMRI. Data were analyzed by one‐way analysis of variance (ANOVA) followed by Tukey post‐hoc; *p < 0.05; *n* = 11–12.

Terminal body weight was significantly greater in 3CAF:2CHOW and CAF groups than controls (**Figure** **9**a; ANOVA: *F*(3, 44) = 4.74, *p* < 0.01; Tukey post‐hoc *p* < 0.05). Similarly, percent fat mass was higher in 3CAF:2CHOW and CAF groups than controls, and in group CAF than group 3CAF:4CHOW (Figure 9b; ANOVA: *F*(3, 44) = 9.45, *p* < 0.01, Tukey post‐hoc *p* < 0.05), with no significant differences in lean mass (Figure 9c; *F*(3, 44) = 1.31, *p* = 0.28). Any form of Caf diet exposure increased retroperitoneal fat mass and plasma leptin, whereas only continuous Caf diet increased body and liver weight, girth, g kg^−1^ retroperitoneal fat and plasma insulin (Supplementary Table S2). Caf diet access had few effects on hippocampal gene expression (Supplementary Figure S1).

### 3.11 Place and Object Recognition

**Figure 10:**
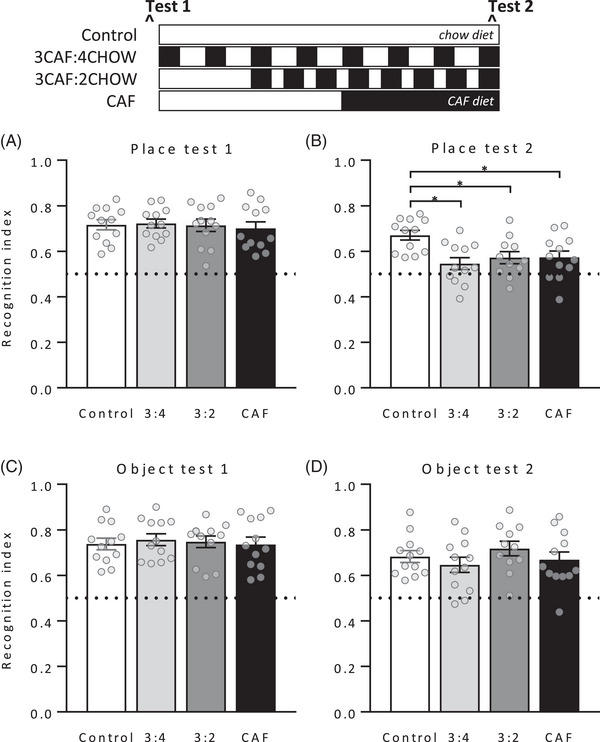
Place and object recognition in Experiment 3. Baseline tests showed no group differences in place A) or object recognition C). At Test 2, held on Caf diet day 22–23, place recognition memory was impaired in all three Caf‐fed groups relative to the control group B), with no difference in object recognition D). *n* = 11–12 per group. Data were analyzed by one‐way analysis of variance (ANOVA) followed by Tukey post‐hoc; *p < 0.05; *n* = 11–12.

Place and object recognition memory did not differ between groups at baseline (**Figure** **10**a,c; both *F* < 1). At test 2, place recognition was impaired in all three Caf‐fed groups relative to controls (Figure 10b; ANOVA *F*(3, 43) = 4.77, *p* < 0.01; Tukey post‐hoc all *p* < 0.05) with no group differences in object recognition (Figure 10d; *F* < 1), or total exploration time in either test (both *F* < 1). Thus, the Caf diet impaired short‐term place recognition memory regardless of whether access was continuous or interspersed by 2‐ or 4‐day chow cycles, indicating that chow cycle length is not a critical mediator of Caf diet‐induced memory impairments under these conditions. The impairment in group 3CAF:4CHOW was unexpected as we have previously found intact place recognition following this schedule^[^ ^31^, ^32^ ^]^ and may relate to the inclusion of pre‐diet baseline tests, the only distinguishing feature of the present study.

### 3.12 Caf Diet Exposure Progressively Impairs Place Recognition Memory

**Figure 11:**
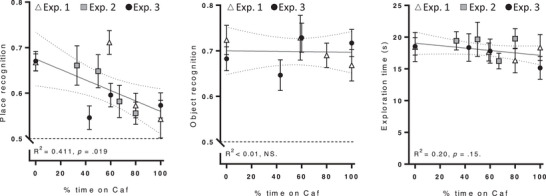
Caf diet exposure is negatively associated with place recognition memory. Group means (*n* = 11–12) for post‐diet place recognition (left), object recognition (middle), and place exploration time (right) are plotted against the percentage of time on Caf diet. A significant negative association was found for place recognition but not for object recognition or total exploration time. There are fewer data points in the middle panel as object recognition was not tested in Experiment 2.

To assess whether cognitive impairment was related to the “concentration” of Caf diet exposure, we expressed each group's cycling schedule as the percentage of time with Caf diet available (e.g., in Experiment 2, group 2CAF:4CHOW = 33% CAF, 4CAF:4CHOW = 50% CAF, etc.), and plotted the relationship between this percentage and cognition (**Figure** **11**). There was a significant negative association between percentage time on Caf diet and place recognition memory (*p* = 0.014, *R* ^2^ = 0.466) but not object recognition memory or total exploration time (*R* ^2^ < 0.01 and *R* ^2^ = 0.20, both *p* > 0.15), suggesting that the relationship was specific to hippocampal‐dependent place recognition memory. Indeed, the correlation between percentage of time on Caf diet and place recognition was somewhat stronger than the correlation between percentage of time on Caf diet and object recognition (Fisher's *r*‐to‐*z* transformation; *z* = −1.61, *p* = 0.054, one‐tailed), but did not differ significantly from the correlation between percentage of time on Caf diet and total exploration time (*z* = −0.69, *p* = 0.245).

### 3.13 Caf Diet Exposure Progressively Alters Gut Microbiota Composition

**Figure 12:**
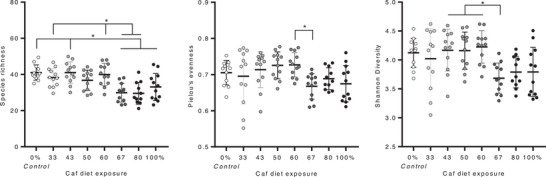
Effects of Caf diet cycling on microbiota alpha diversity. Relative to control rats fed chow, Margalef's richness (left) was significantly reduced by higher percent time exposed to the Caf diet, with the 50% Caf group not significantly different to any other group. The only significant pairwise difference in Pielou's evenness (middle) was between 60% and 67% Caf groups, whereas Shannon diversity (right) differed between groups fed 43%–60% Caf relative to 67% Caf diet. Data were analyzed by one‐way analysis of variance (ANOVA) followed by Tukey post‐hoc tests; *p < 0.05; *n* = 11–12. Groups 33%, 50%, 67%, and 80% Caf from Experiment 2; Control, 43%, 60%, and 100% Caf from Experiment 3.

Analyses of fecal microbiota composition in Experiments 2 and 3 (*N* = 95) showed that the percentage of Caf diet exposure was significantly related to Margalef's richness (**Figure** **12**; *F*(7, 87) = 7.94, *p* < 0.001), Pielou's evenness (*F*(7, 87) = 2.79, *p* = 0.012) and Shannon diversity (*F*(7, 87) = 4.24, *p* < 0.001). Relative to control, species richness and Shannon diversity were significantly reduced in 67%, 80%, and 100% Caf diet groups (Tukey post‐hoc, all *p* < 0.025), but not 33%, 43%, 50%, and 60% Caf diet groups (all *p* < 0.63). A 67% Caf diet reduced evenness and Shannon diversity relative to 60% and 50% Caf groups, and also reduced Shannon diversity relative to the 43% Caf group (all *p* < 0.042). At the group level, percent time on the Caf diet was negatively associated with mean richness (*r*(6) = −0.724, *p* = 0.042) but not Pielou's evenness *r*(6) = −0.461, *p* = 0.250) or Shannon diversity (*r*(6) = −0.612, *p* = 0.107).

**Figure 13:**
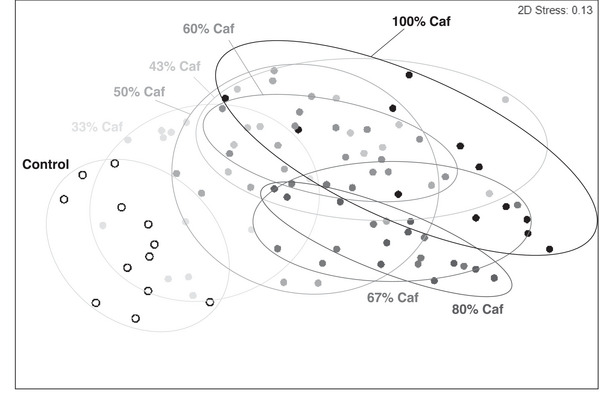
Nonmetric multidimensional scaling plot of gut microbiota composition in rats exposed to various schedules of cycling Caf diet. Relative to control rats fed chow (unfilled circles, left), increasing the proportion of Caf diet access (% days with Caf diet available) progressively shifted microbiota composition toward that of rats fed Caf diet continuous (100% Caf diet, top right). Permutational multivariate analysis of variance (PERMANOVA) analysis and pairwise comparisons indicated that all groups differed significantly except for three pairs of groups given cycling Caf (67% versus 80%, 33% versus 50%, 43% versus 60% Caf diet). Data points represent individual rats and proximity reflects microbiota similarity. Plot derived from a Bray–Curtis similarity index at the OTU level, *n* = 11–12 per group.

**Figure** **13** shows a nonmetric multidimensional scaling plot of microbiota composition, analyzed by PERMANOVA with group as a fixed factor (eight levels) and cage as a random factor (32 levels, nested in group). There were significant group differences (997 permutations, pseudo‐*F*(7, 63) = 3.78, *p* = 0.001) and cage effects (996 permutations, *df* = 24, pseudo‐*F* = 1.31, *p* = 0.001). Follow‐up pairwise comparisons applying Monte Carlo simulations showed that any Caf diet exposure shifted microbiota composition relative to controls (all *p* < 0.007) and, conversely, any reduction in the proportion of time on Caf diet significantly altered microbiota composition relative to continuous Caf (all *p* < 0.038). The only nonsignificant differences were group 33% versus 50% Caf (*p* = 0.054), group 67% versus 80% Caf (*p* = 0.657) and groups 43% versus 60% Caf (*p* = 0.428). PERMDISP analysis found no differences in sample dispersion across groups (999 permutations; *F*(7, 87) = 1.021, *p* = 0.53).

### 3.14 Differences in Place Recognition and Whole‐Body Adiposity Predict Global Microbiota Composition

Distance‐based linear modeling was used to examine how variability in the OTU similarity matrix was related to place recognition at the final test, total exploration time in this test (a control for general activity), fat mass as percentage of body weight, net lean mass (a measure of growth), and liver mass as percentage of body weight. Place recognition memory was the strongest predictor of variability in the OTU matrix (pseudo‐*F* = 2.907, *p* = 0.002), followed by percent fat mass (pseudo‐*F* = 2.216, *p* = 0.01) and weight‐adjusted liver mass (pseudo‐*F* = 1.995, *p* = 0.023). The effect of lean mass was marginal (pseudo‐*F* = 1.59, *p* = 0.052) while total exploration time was a nonsignificant predictor (pseudo‐*F* = 0.88, *p* = 0.541). Supplementary Figures S2–S4 show additional analyses identifying 20 OTUs that were progressively enriched or depleted by increasing Caf diet exposure, and of the impact of switching to chow diet for 4 days in Experiment 2. To determine whether any of the 20 taxa associated with Caf diet exposure (Supplementary Figure S4) were also linked to cognitive impairment, we correlated their relative abundance with place recognition memory and applied a Benjamini–Hochberg correction. This revealed that place recognition correlated positively with the relative abundance of OTU14_Phascolarctobacterium (*r* = 0.332, *p* = 0.0018, *q* = 0.1) and negatively with the relative abundance of OTU2_Bacteroidetes (*r* = −0.264, *p* = 0.001, *q* = 0.1). BLAST analyses indicated that OTU14_Phascolarctobacterium shared 97.62% similarity with *Phascolarctobacterium faecium* and OTU2_Bacteroides shared 100% similarity with *Bacteroides (Phocaeicola) vulgatus*.

## 4 Discussion

People often alternate between healthy and unhealthy diets, yet the effects of diet cycling on cognition and gut microbiota are not well understood. Here, we show that cycling between chow and an obesogenic diet of processed foods eaten by people impairs short‐term place recognition memory and alters gut microbiota composition in an apparently “dose‐dependent” fashion, with greater effects produced by increasing ratios of Caf:Chow diet. Differences can be attributed to the pattern of obesogenic diet access per se because we matched total days of Caf diet across groups in each experiment. Experiments tested the importance of three key variables in diet cycling: we first found time‐dependent effects of withdrawal from the Caf diet on place recognition memory (Experiment 1), with intact performance in rats withdrawn from the Caf diet for 11 but not 4 days. We next showed that manipulating Caf cycle length led to graded changes in place recognition memory (Experiment 2), with poorer performance following 16‐day than 2‐day cycles, and intermediate performance following 4‐ and 8‐day cycles. Conversely, there were no differences in Caf diet‐induced memory impairments when the chow cycle length was manipulated (Experiment 3). Together, results suggest that Caf cycle length is the critical determinant of diet‐induced cognitive impairment in diet cycling.

Caf diet exposure led to robust metabolic effects, increasing body weight gain and adiposity relative to chow‐fed controls even over the relatively short time courses studied here (16–24 days access), and despite the pronounced hypophagia in cycled groups during chow cycles. In Experiment 2, even 2 days of Caf diet interleaved with 4 days of chow over 24 days was sufficient to increase adiposity in the 2CAF:4CHOW group. The final experiment showed that although Caf diet cycling led to stepwise increases in body weight, as shown previously,^[^ ^12^, ^31^, ^32^, ^36^ ^]^ most metabolic measures were increased by any form of Caf diet access. Indeed, the metabolic effects of the diet cycling schedules tested here generally did not align with cognitive effects. For example, removing Caf diet for 11 days improved place recognition even though adiposity remained higher than in chow‐fed controls (Experiment 1), and increasing Caf cycle length exacerbated the impairment in place recognition memory despite a comparable increase in adiposity in all groups relative to baseline (Experiment 2). Thus, the effects of the diet cycling schedule on cognition did not require group differences in adiposity.

Our finding that gut microbiota composition is progressively altered by obesogenic diet exposure is consistent with previous work showing rapid shifts in microbiota composition in humans following a change in diet^[^ ^29^ ^]^ or in rodents given brief daily access to palatable foods.^[^ ^37^ ^]^ Distance‐based linear modeling indicated that the cognitive and metabolic measures most strongly affected by diet cycling – that is, place recognition memory, total adiposity, and liver mass – were also the strongest predictors of global microbiota composition, whereas lean mass and total exploration (a proxy for total activity) were not associated with the microbiota. Overall, these analyses suggest that the adverse effects of diet cycling on cognitive, metabolic, and microbial measures were closely related. Higher Caf diet concentrations progressively reduced the relative abundance of five *Porphyromonadaceae* taxa, which is depleted in children with obesity,^[^ ^38^ ^]^ associated with leanness in older adults,^[^ ^39^ ^]^ and increased by liquid sugar in rats.^[^ ^40^ ^]^ There were also progressive reductions in two *Prevotella* taxa with higher Caf diet concentrations, in agreement with results from rodent high‐fat diet models.^[^ ^41^ ^]^ *Prevotella* abundance, which may indicate adherence to a plant‐based diet in people,^[^ ^42^ ^]^ has been positively associated with weight loss in overweight adults across a 6‐week diet intervention.^[^ ^43^ ^]^ Finally, Caf diet concentration was also positively associated with the relative abundance of two *Bacteroides* spp., consistent with other rodent models of obesogenic diet consumption.^[^ ^44^, ^45^, ^46^ ^]^

To further link diet‐induced microbiota changes to the cognitive phenotype, we tested for associations between the 20 OTUs altered by Caf diet exposure and place recognition memory. Analyses revealed that place recognition memory was positively associated with the relative abundance of OTU14_Phascolarctobacterium, putatively identified as *P. faecium*, and negatively associated with the relative abundance of OTU2_Bacteroides, putatively identified as *B. (Phocaeicola) vulgatus*. The former result is consistent with a study showing that *Phascolarctobacterium* abundance correlated positively with scores on the Montreal Cognitive assessment in patients with ischemic stroke^[^ ^47^ ^]^; however, another meta‐analysis found that patients on the Alzheimer's disease spectrum displayed greater abundance of *Phascolarcobacterium* at the genus level.^[^ ^48^ ^]^ Evidence for an association with cognition is also mixed for *B. vulgatus*, which has been associated with intact cognition in older adults,^[^ ^49^ ^]^ but found in another study to be enriched in children with autism spectrum disorder.^[^ ^50^ ^]^ Therefore, more work is needed to clarify how diet‐induced changes in the abundance of these bacteria may moderate cognitive function.

Intermittent access schedules can induce “binge‐like” consumption of sucrose solutions,^[^ ^51^, ^52^, ^53^ ^]^ high‐fat foods,^[^ ^54^ ^]^ or the cafeteria‐style diet used here.^[^ ^12^ ^]^ Unlike these studies, 24‐h energy intake on Caf diet in the present experiments did not differ between cycled and continuous Caf groups, though intake in the first hours of access may have been elevated, as reported in previous intermittent access models.^[^ ^53^ ^]^ However, cycled Caf groups suppressed energy intake during chow cycles, consuming approximately half as much chow as controls, consistent with our prior work.^[^ ^12^ ^]^ A novel result when Caf cycle length was varied (Experiment 2) was that the reduction in chow intake was proportional to the preceding time on Caf diet, with longer Caf cycles leading to a greater suppression in consumption. Reduced energy intake during chow cycles was likely due to negative incentive contrast (i.e., the lower palatability of chow) and possibly stress‐induced suppression of appetite. Withdrawal from palatable diets increases anxiety‐like behavior^[^ ^8^ ^]^ and upregulates stress markers in the amygdala^[^ ^55^ ^]^ and hypothalamus.^[^ ^56^ ^]^ Conversely, acute stress can also enhance intake of palatable food provided on an intermittent access schedule,^[^ ^57^ ^]^ a positive incentive contrast effect which may have increased Caf intake in the cycled groups immediately following a chow cycle. Finally, while food was always available in these experiments, our results are interesting in light of a recent study showing that chronic exposure to an unpredictable food schedule produced greater future weight gain and higher progressive ratio breakpoints for food reward in rats.^[^ ^58^ ^]^ Thus, to the extent the Caf cycling schedules used here were unpredictable, these data imply that diet cycling may confer risk of overeating and greater weight gain if an obesogenic diet is subsequently available ad libitum.

Analyses of gene expression in Experiment 3 found few changes in hippocampal neuroinflammatory (*Il6*, *Il1b*, *Ikbkb*, *Aif1*), neurotrophic (*Bdnf*), or blood–brain barrier integrity markers (*Ocln*, *Cldn5*, *Glut1*) following continuous or cycling Caf exposure, in contrast to our previous studies.^[^ ^31^, ^59^ ^]^ The absence of such effects here may relate to time spent on the diet, which differed from these previous studies. Indeed, effects of obesogenic diets on the brain are time‐dependent and region‐specific. For example, hypothalamic oxidative stress gene expression is altered within hours of exposure to a western‐style diet^[^ ^60^ ^]^ and hypothalamic inflammation was shown to increase in rats after 1–3 days or 4 weeks of a high‐fat diet, but not 1 or 2 weeks.^[^ ^61^ ^]^ Dietary effects may also vary between cell types, for example, greater gene expression changes have been observed in astrocytes than microglia or neurons after relatively short (5 day) exposure to a high‐fat, high‐sugar diet in mice.^[^ ^62^ ^]^ In contrast to such effects, extended access to obesogenic diets appears necessary to increase blood‐brain barrier permeability.^[^ ^63^ ^]^

### 4.1 Future Directions

In addition to the short‐term recognition memory tests used here, it will be important to test whether Caf diet cycling exerts graded effects on cognitive processes that recruit other brain regions, including measures of executive function such as reversal learning^[^ ^64^ ^]^ and delay discounting,^[^ ^65^ ^]^ mediated by the prefrontal cortex, and reward‐related behaviors of food‐seeking shown to be dysregulated by junk food consumption.^[^ ^66^, ^67^, ^68^, ^69^, ^70^ ^]^ The relatively brief chow cycles tested here (2–4 days) led to minimal changes in body weight and thus modeled short‐term *diet* cycling rather than *weight* cycling. It will be informative to test whether diet cycling schedules that produce appreciable weight loss protect against cognitive impairment, as weight loss is associated with improved cognition in people with obesity.^[^ ^71^ ^]^ A related point is that our experiments used young adult rats that began Caf diet cycling while still growing, raising the possibility that the groups starting diet cycling when younger had some protection against metabolic impairments relative to those introduced to Caf diet several weeks later. Thus, the effects of Caf diet cycling on metabolic parameters should be studied in older rats when body weight has stabilized. That said, earlier onset of Caf diet exposure might be expected to increase vulnerability to cognitive impairment^[^ ^72^ ^]^ but this was not supported by the present data. Finally, although our data indicate that leaner schedules of access to an unhealthy diet were protective, these benefits may wane over longer‐term exposure as effects of the diet per se accrue.

In sum, the present study demonstrates progressive changes in spatial memory and gut microbiota composition produced by cycling access to a high‐fat, high‐sugar diet. In light of high discretionary food intake in human populations, the Caf cycled groups tested here appear to be a relevant model of modern eating habits for many people. Our results suggest that even subtle shifts in dietary habits are likely to confer changes in cognitive and metabolic health over the longer term.

## Conflict of Interest

The authors declare no conflict of interest.

## Author Contributions

Experimental design M.D.K., R.F.W., and M.J.M.; data acquisition M.D.K., K.H., and S.L.; data analysis M.D.K., K.H., and N.K.; initial draft M.D.K.; all authors contributed to revisions; and funding M.J.M., R.F.W.

## Supporting information

Supplementary Information

## Data Availability

The data that support the findings of this study are available from the corresponding author upon reasonable request.
