# Supplementary material for: Obesogenic Diet Cycling Produces Graded Effects on Cognition and Microbiota Composition in Rats
Source: Mol Nutr Food Res. 2023 May 5;67(12):2200809. doi: 10.1002/mnfr.202200809 (PMC10909530; doi:10.1002/mnfr.202200809)
Supplement: Supplementary file 1 — Supplementary Information [file MNFR-67-2200809-s001.pdf]

Supplementary materials

Supplementary Table S1. Experiment 2 endpoint measures (mean ± SEM). There were no significant group differences on any parameters. [1]Liver scores analysed by nonparametric Kruskal-Wallis and Kolmogorov-Smirnov tests.

Measure	2CAF:4CHOW	4CAF:4CHOW	8CAF:4CHOW	16CAF:4CHOW	ANOVA *p*
Body weight (g)	549.2 (11.6)	556.7 (10.5)	538.8 (10.8)	547.2 (9.4)	0.70
Nasoanal length (cm)	24.83 (0.18)	24.83 (0.24)	24.88 (0.20)	25.08 (0.14)	0.76
Girth (cm)	18.92 (0.3)	19.08 (0.2)	18.63 (0.3)	18.88 (0.2)	0.62
Liver score[1] (0-3)	1.67 (0.19)	2.33 (0.22)	2.25 (0.13)	2.17 (0.17)	0.07
Liver weight (g)	17.32 (0.65)	17.66 (0.41)	16.54 (0.44)	16.97 (0.54)	0.47
Liver weight (% BW)	3.15 (0.08)	3.17 (0.04)	3.07 (0.03)	3.10 (0.06)	0.54
RP WAT (g)	9.40 (0.90)	10.48 (0.61)	8.77 (0.62)	8.87 (0.93)	0.40
Gonadal WAT (g)	9.62 (0.68)	10.07 (0.73)	8.21 (0.53)	9.02 (0.72)	0.25
Total WAT (g/kg)	34.3 (2.2)	36.7 (1.8)	31.4 (1.8)	32.5 (2.6)	0.33
Blood glucose (mM)	9.3 (0.3)	9.6 (0.5)	8.7 (0.2)	8.5 (0.2)	0.10
Plasma insulin (ng/ml)	0.65 (0.05)	0.78 (0.10)	0.59 (0.03)	0.65 (0.04)	0.16

Supplementary Table S2. Experiment 3 endpoint measures (mean ± SEM). Significant ANOVA main effects (right column) were followed by post-hoc Tukey comparisons; groups not sharing a letter differ significantly at $p < .05$. [1]Liver scores were analysed by nonparametric Kruskal-Wallis and Kolmogorov-Smirnov tests.

Measure	Chow	3CAF:4CHOW	3CAF:2CHOW	CAF	ANOVA *p*
Body weight (g)	533.8^{A} (19.7)	591.7^{AB} (19.4)	607.9^{B} (18.0)	634.1^{B} (20.9)	**0.006**
Nasoanal length (cm)	25.2 (0.3)	25.3 (0.3)	25.4 (0.2)	25.7 (0.2)	0.078
Girth (cm)	18.8^{A} (0.3)	20.0^{AB} (0.5)	20.6^{B} (0.4)	20.8^{B} (0.5)	**0.012**
Liver score[1] (0-3)	0.9^{A} (0.2)	2.3^{B} (0.3)	2.8^{B} (0.1)	2.8^{B} (0.1)	**< .01**
Liver weight (g)	18.6^{A} (1.0)	21.4^{AB} (1.2)	22.1^{AB} (1.0)	227^{B} (0.9)	**0.040**
Liver weight (% BW)	3.30 (0.09)	3.44 (0.09)	3.46 (0.08)	3.41 (0.07)	0.545
Retroperitoneal WAT (g)	8.14^{A} (0.8)	11.8^{B} (1.3)	11.8^{B} (0.8)	13.8^{B} (0.8)	**0.001**
Retroperitoneal WAT (g/kg)	15.06^{A} (1.2)	19.40^{AB} (1.7)	19.35^{AB} (1.2)	21.77^{B} (0.9)	**0.005**
Blood glucose (mmol/L)	9.99 (0.32)	9.85 (0.30)	10.08 (0.31)	10.01 (0.68)	0.985
Plasma insulin (ng/ml)	0.41^{A} (0.07)	0.86^{AB} (0.16)	0.93^{AB} (0.13)	1.38^{B} (0.31)	**0.013**
Plasma leptin (ng/ml)	3.71^{A} (0.35)	7.23^{B} (0.96)	7.77^{B} (0.83)	8.70^{B} (0.98)	**0.001**

Supplementary Table S3. Taq assay probe identifiers assessed in dorsal hippocampus (Experiment 3)

Gene	Unique assay identifier
Allograft inflammatory factor 1 (*Aif1*)	Rn00574125_g1
Brain-derived neurotrophic factor (*Bdnf*)	Rn02531967_s1
Claudin-5 (*Cldn5*)	Rn01753146_s1
Glial fibrillary acidic protein (*Gfap*)	Rn01253033_m1
Glucose transporter 1 (*Glut1*)	Rn01417099_m1
Hypoxanthine phosephoribosyltransferase 1 (*Hprt1*)	Rn01527840_m1
Inhibitor of nuclear factor kappa B kinase subunit beta (*Ikbkb*)	Rn00584379_m1
Insulin receptor (*Insr*)	Rn00690703_m1
Interleukin-1 beta (*Il1B*)	Rn00580432_m1
Interleukin-6 (*Il6*)	Rn01410330_m1
Occludin (*Ocln*)	Rn00580064_m1
Tyrosine 3-monooxygenase/tryptophan 5-monooxygenase activation protein zeta (*Ywhaz*)	Rn00755072_m1

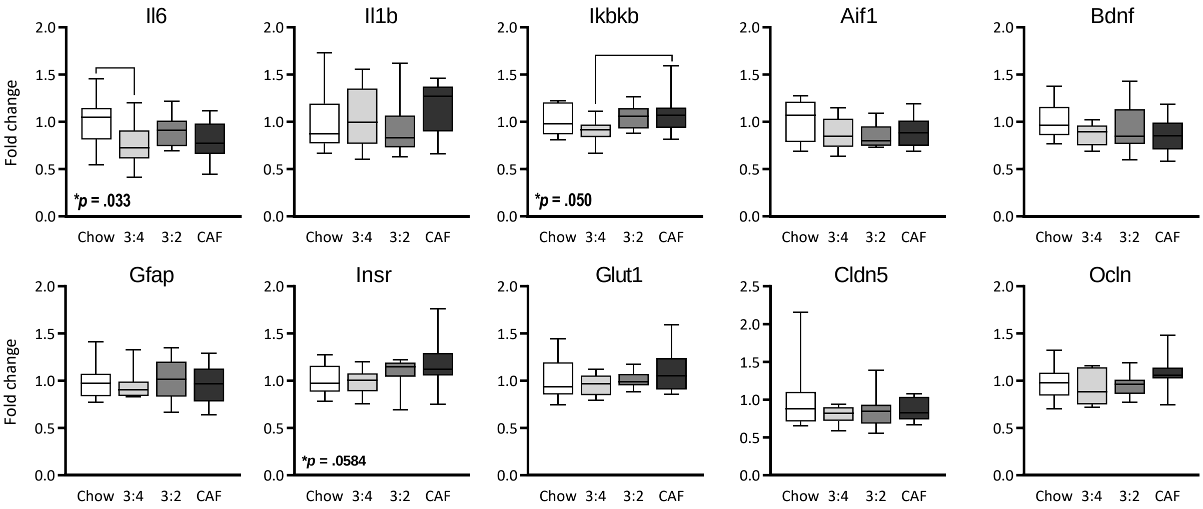

Supplementary Figure S1. Relative expression of inflammatory, neurotrophic and blood-brain barrier integrity genes in dorsal hippocampus. Data shown as box and whisker plots with inter-quartile range (minimum-maximum values). P-values at inset show significant overall ANOVA effect; brackets show significant post-hoc pairwise comparisons applying the Tukey HSD correction. *n* = 11/group. In Experiment 3, the dorsal hippocampus was dissected on ice and snap-frozen in liquid nitrogen. Hippocampal RNA was extracted using a

standard protocol following homogenization in TRI-Reagent® (Sigma-Aldrich, Australia, T9424). After measuring concentration and quality (DeNovix Inc., USA), RNA was reverse transcribed to cDNA (High-capacity reverse transcriptase kit; ThermoFisher Scientific, USA; HCAPRT) after treatment with DNase I (Sigma-Aldrich, Australia, AMPD1), according to manufacturer protocols. TaqMan inventoried gene expression assays (Life Technologies Pty Ltd, Australia, Supplementary Table S3) were used to assess gene expression in duplicate. Expression of genes of interest were normalised to the geometric mean of Ywhaz and Hprt1. The ddCT method was used to calculate relative gene expression normalised to an independent calibrator[73].

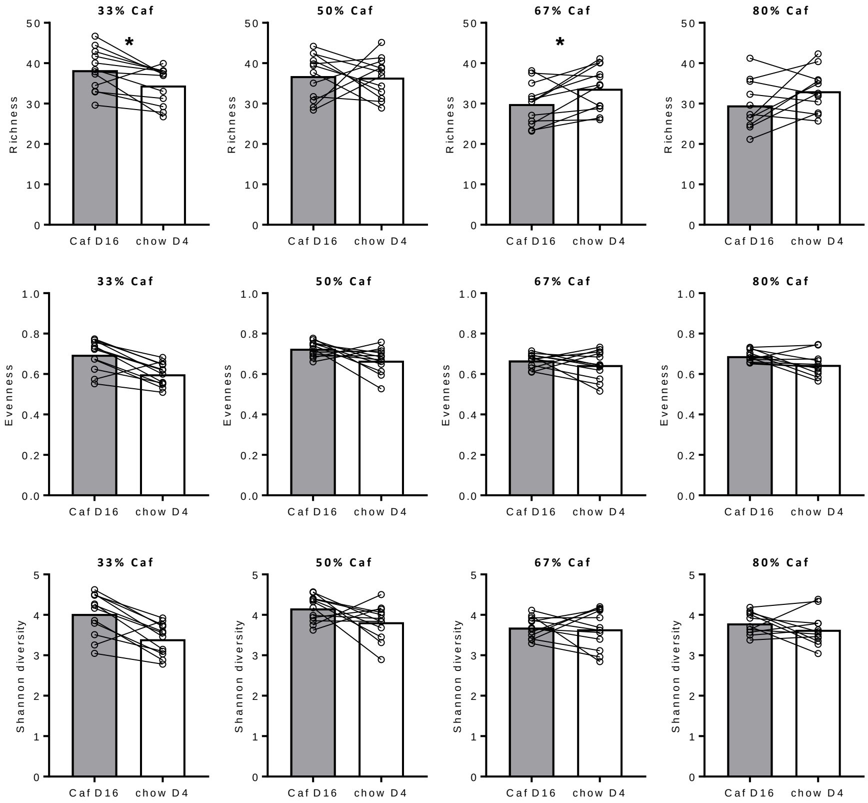

Supplementary Figure S2. Effects of an acute diet switch on faecal microbiota alpha diversity. A second faecal sample was collected from rats in Experiment 2 (33%, 50%, 67% and 80% CAF groups) after the final 4-day chow cycle. Data analysed in 4 x (2) mixed-ANOVA. A significant group x time interaction for Margalef's richness (top) ($F(3, 43) = 3.79$, $p = .017$) was driven by richness decreasing in the 2CAF:4CHOW group ($p = .01$) and increasing in the 8CAF:4CHOW group ($p = .033$). The switch to chow significantly decreased Pielou's evenness ($F(1, 43) = 24.24$, $p < .001$) and Shannon diversity ($F(1, 43) = 13.23$, $p = .001$) with no group x time interactions ($F(3, 43) = 2.06$, $p = .12$ and $F(3, 43) = 2.68$, $p = .059$, respectively. There were no significant differences in richness, evenness or Shannon diversity at the second timepoint when all rats were consuming chow (largest $F(3, 43) = 1.219$, $p = .31$). *n*=11-12. *$p < .05$ for within-group change. Values obtained on Caf day 16 were also used in the combined analysis shown in Figure 11. Group 33% Caf = 2CAF:4CHOW; 50% Caf = 4CAF:4CHOW; 67% Caf = 8CAF:4CHOW; 80% Caf = 16CAF:4CHOW.

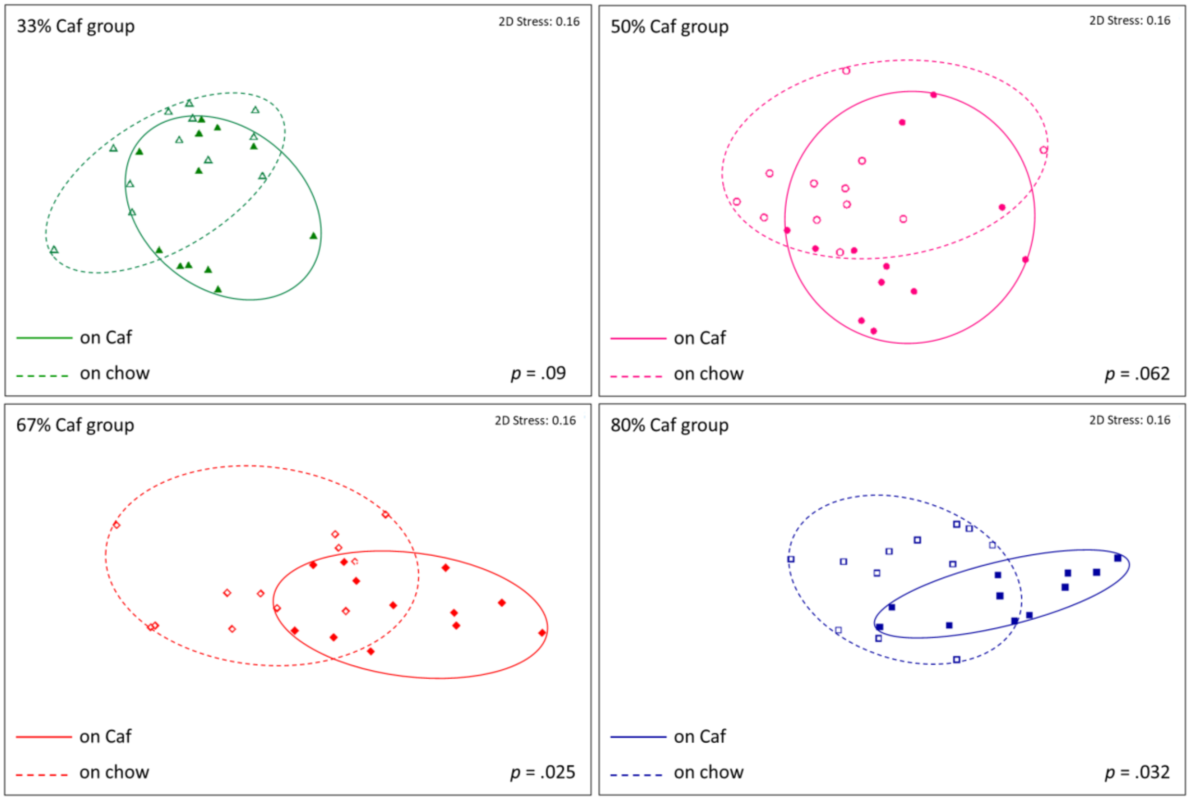

Supplementary Figure S3. Non-metric multidimensional scaling plots showing shifts in global microbiota composition following an acute 4-day switch from Caf to chow diet. The diet switch significantly changed composition in groups with >50% Caf diet exposure (67% and 80% Caf groups, bottom panels) with non-significant trends for groups with ≤50% Caf diet exposure (33% and 50% groups, top panels). Data analysed by PERMANOVA with group (4 levels) and time (2 levels) as fixed factors, and cage, nested in group, as a random factor (16 levels). Analyses revealed a group x time interaction (996 permutations, pseudo-F(3, 63) = 1.70, p = .022) and significant effects of group (999 permutations, pseudo-F(3, 63) = 2.59, p = .001), time (999 permutations, pseudo-F(1, 63) = 10.73, p = .001), cage (995 permutations, pseudo-F(12, 63) = 1.922, p = .001) with no cage x time interaction (996 permutations, pseudo-F(12,63) = .87, p = .981). Pairwise tests found that the 4-day switch to chow significantly altered composition in group 67% Caf (p = .025) and group 80% Caf (p = .032) but not in group 33% Caf (p = .09) or 50% Caf (p = .062). Data on Caf day 16 are also presented in the combined analysis shown in Figure 12.

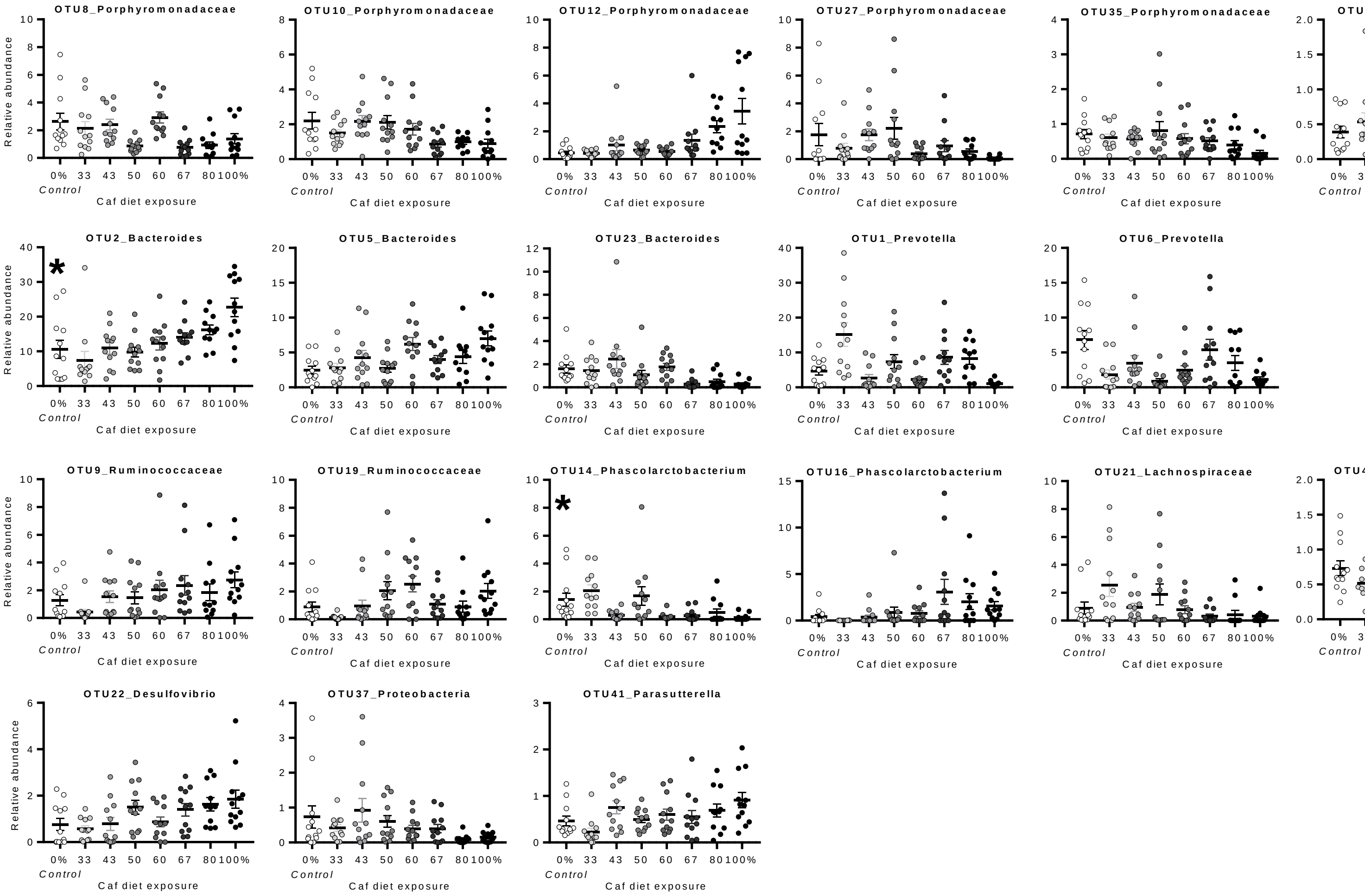

Supplementary Figure S4. To identify bacterial taxa that were progressively altered by Caf diet concentration, the relative abundance (% total OTUs) of the top 50 OTUs was regressed against 3 predictor variables: “% time on Caf diet”, “home cage”, and “Experiment” (2 or 3). OTUs were selected for further examination if the overall regression model was significant and % time on Caf diet was significant. Relative abundance plots are shown for 20 OTUs that met these criteria after applying a Benjamini-Hochberg correction (FDR = 0.05). Of the 11 OTUs from phylum *Bacteroidetes*, higher Caf diet concentrations reduced the relative abundance of 5/6 *Porphorymonadaceae* taxa (top row) and two *Prevotella* taxa (OTU1, OTU6), with mixed effects for *Bacteroides*. Within phylum *Firmicutes*, higher Caf diet concentrations increased and reduced the relative abundance of *Ruminococcaceae* and *Lachnospiraceae* taxa, respectively. 33%, 50%, 67% and 80% Caf groups are from Experiment 2; control, 43%, 60% and 100% Caf groups are from Experiment 3. *OTU2 and OTU14 correlated significantly with place recognition memory (see main text for details).
